# Supplementary material for: Albumin-Bound Fatty Acids Modulate Endogenous Angiotensin-Converting Enzyme (ACE) Inhibition
Source: Biomedicines. 2026 Jan 4;14(1):103. doi: 10.3390/biomedicines14010103 (PMC12838124; doi:10.3390/biomedicines14010103)
Supplement: Supplementary file 1 [file biomedicines-14-00103-s001.zip › Supplementary Figure S1.pdf]

Supplementary Figure S1 - Plasmid sequence of ′CS-Y4341-I03-02′

aacccagctttcttgtacaaagtggtgatgccatggatccggaaatcaaaggcctacgtcgacgagctcaactagtgcggccgctgtcgagaagtac
tagaggatcataatcagccataccacatttgtagaggttttacttgctttaaaaaacctcccacacctccccctgaacctgaaacataaaatgaatgcaat
tgttgttgttaacttgtttattgcagcttataatggttacaaataaagcaatagcatcacaaatttcacaaataaagcatttttttcactgcattctagttgtg
gtttgtccaaactcatcaatgtatcttatcatgtctggatctgatcactgcttgagcctaggagatccgaaccagataagtgaaatctagttccaaactatt
ttgtcatttttaattttcgtattagcttacgacgctacacccagttcccatctattttgtcactcttccctaaataatccttaaaaactccatttccacccctccca
gttcccaactattttgtccgcccacagcggggcatttttcttcctgttatgtttttaatcaaacatcctgccaactccatgtgacaaaccgtcatcttcggcta
ctttttctctgtcacagaatgaaaatttttctgtcatctcttcgttattaatgtttgtaattgactgaatatcaacgcttatttgcagcctgaatggcgaatggg
acgcgccctgtagcggcgcattaagcgcggcgggtgtggtggttacgcgcagcgtgaccgctacacttgccagcgccctagcgcccgctcctttcgc
tttcttcccttcctttctcgccacgttcgccggctttccccgtcaagctctaaatcgggggctccctttagggttccgatttagtgctttacggcacctcgacc
ccaaaaaacttgattagggtgatggttcacgtagtgggccatcgccctgatagacggtttttcgccctttgacgttggagtccacgttctttaatagtgg
actcttgttccaaactggaacaacactcaaccctatctcggtctattcttttgatttataagggattttgccgatttcggcctattggttaaaaaatgagctg
atttaacaaaaatttaacgcgaattttaacaaaatattaacgtttacaatttcaggtggcacttttcggggaaatgtgcgcggaacccctatttgtttatttt
tctaaatacattcaaatatgtatccgctcatgagacaataaccctgataaatgcttcaataatattgaaaaaggaagagtatgagtattcaacatttccgt
gtcgcccttattcccttttttgcggcattttgccttcctgtttttgctcacccagaaacgctggtgaaagtaaaagatgctgaagatcagttgggtgcacga
gtgggttacatcgaactggatctcaacagcggtaagatccttgagagttttcgccccgaagagcgttttccaatgatgagcacttttaaagttctgctat
gtggcgcggtattatcccgtattgacgccgggcaagagcaactcggtcgccgcatacactattctcagaatgacttggttgagtactcaccagtcaca
gaaaagcatcttacggatggcatgacagtaagagaattatgcagtgctgccataaccatgagtgataacactgcggccaacttacttctgacaacgat
cggaggaccgaaggagctaaccgcttttttgcacaacatgggggatcatgtaactcgccttgatcgttgggaaccggagctgaatgaagccatacca
aacgacgagcgtgacaccacgatgcctgtagcaatggcaacaacgttgcgcaaactattaactggcgaactacttactctagcttcccggcaacaatt
aatagactggatggaggcggataaagttgcaggaccacttctgcgctcggcccttccggctggctggtttattgctgataaatctggagccggtgag
cgtgggtctcgcggtatcattgcagcactggggccagatggtaagccctcccgtatcgtagttatctacacgacggggagtcaggcaactatggatg
aacgaaatagacagatcgctgagataggtgcctcactgattaagcattggtaactgtcagaccaagtttactcatatatactttagattgatttaaaactt
catttttaatttaaaaggatctaggtgaagatcctttttgataatctcatgaccaaaatcccttaacgtgagttttcgttccactgagcgtcagaccccgta
gaaaagatcaaaggatcttcttgagatcctttttttctgcgcgtaatctgctgcttgcaaacaaaaaaaccaccgctaccagcggtggtttgtttgccgg
atcaagagctaccaactctttttccgaaggtaactggcttcagcagagcgcagataccaaatactgtccttctagtgtagccgtagttaggccaccactt
caagaactctgtagcaccgcctacatacctcgctctgctaatcctgttaccagtggctgctgccagtggcgataagtcgtgtcttaccgggttggactca
agacgatagttaccggataaggcgcagcggtcgggctgaacggggggttcgtgcacacagcccagcttggagcgaacgacct
acaccgaactgagatacctacagcgtgagcattgagaaagcgccacgcttcccgaagggagaaaggcggacaggtatccggtaagcggcaggg
tcggaacaggagagcgcacgagggagcttccagggggaaacgcctggtatctttatagtcctgtcgggtttcgccacctctgacttgagcgtcgattt
ttgtgatgctcgtcaggggggcggagcctatggaaaaacgccagcaacgcggcctttttacggttcctggccttttgctggccttttgctcacatgttctt
tcctgcgttatcccctgattctgtggataaccgtattaccgcctttgagtgagctgataccgctcgccgcagccgaacgaccgagcgcagcgagtcagt
gagcgaggaagcggaagagcgcctgatgcggtattttctccttacgcatctgtgcggtatttcacaccgcagaccagccgcgtaacctggcaaaatc
ggttacggttgagtaataaatggatgccctgcgtaagcgggtgtgggcggacaataaagtcttaaactgaacaaaatagatctaaactatgacaata
aagtcttaaactagacagaatagttgtaaactgaaatcagtccagttatgctgtgaaaaagcatactggacttttgttatggctaaagcaaactcttcatt
ttctgaagtgcaaattgcccgtcgtattaaagaggggcgtggccaagggcatggtaaagactatattcgcggcgttgtgacaatttaccgaacaactc
cgcggccgggaagccgatctcggcttgaacgaattgttaggtggcggtacttgggtcgatatcaaagtgcatcacttcttcccgtatgcccaactttgt
atagagagccactgcgggatcgtcaccgtaatctgcttgcacgtagatcacataagcaccaagcgcgttggcctcatgcttgaggagattgatgagc
gcggtggcaatgccctgcctccggtgctcgccggagactgcgagatcatagatatagatctcactacgcggctgctcaaacctgggcagaacgtaa
gccgcgagagcgccaacaaccgcttcttggtcgaaggcagcaagcgcgatgaatgtcttactacggagcaagttcccgaggtaatcggagtccgg
ctgatgttgggagtaggtggctacgtctccgaactcacgaccgaaaagatcaagagcagcccgcatggatttgacttggtcagggccgagcctacat
gtgcgaatgatgcccatacttgagccacctaactttgttttagggcgactgccctgctgcgtaacatcgttgctgctgcgtaacatcgttgctgctccata
acatcaaacatcgacccacggcgtaacgcgcttgctgcttggatgcccgaggcatagactgtacaaaaaaacagtcataacaagccatgaaaaccg
ccactgcgccgttaccaccgctgcgttcggtcaaggttctggaccagttgcgtgagcgcatacgctacttgcattacagtttacgaaccgaacaggctt
atgtcaactgggttcgtgccttcatccgtttccacggtgtgcgtcacccggcaaccttgggcagcagcgaagtcgaggcatttctgtcctggctggcga
acgagcgcaaggtttcggtctccacgcatcgtcaggcattggcggccttgctgttcttctacggcaaggtgctgtgcacggatctgccctggcttcagg
agatcggaagacctcggccgtcgcggcgcttgccggtggtgctgaccccggatgaagtggttggcatcctcggttttctggaaggcgagcatcgttt
gttcgcccaggactctagctatagttctagtggttggctacgtatactccggaatattaatagatcatggagataattaaagtgataaccatctcgcaaa
taaataagtattttactgttttcgtaacagttttgtaataaaaaaacctataaatattccggattattcataccgtcccaccatcgggcgcggatctcggtcc
gaaattcGAAGGAATTCGGTACCATGGCGCCGCCTCCGGCCGCCGCGGCCCCGGCCTGCTGCTG

CCCCTGCCCCTGCTGCTGCTGCTGCCCCCCCAGCCCGCCCTGGCCCACCACCACCACCACCAC
TCCGGCGAGAACCTGTACTTCCAGGGCCTGGACCCCGGCCTGCAGCCCGGCAACTTCTCCGCC
GACGAGGCCGGCGCCCAGCTGTTCGCCCAGTCCTACAACTCCTCCGCCGAGCAGGTGCTGTTC
CAGTCCGTGGCCGCCTCCTGGGCCCACGACACCAACATCACCGCCGAGAACGCCCGCCGCCA
GGAGGAGGCCGCCCTGCTGTCCCAGGAGTTCGCCGAGGCCTGGGGCCAGAAGGCCAAGGAG
CTGTACGAGCCCATCTGGCAGAACTTCACCGACCCCCAGCTGCGCCGCATCATCGGCGCCGTG
CGCACCCTGGGCTCCGCCAACCTGCCCCTGGCCAAGCGCCAGCAGTACAACGCCCTGCTGTC
CAACATGTCCCGCATCTACTCCACCGCCAAGGTGTGCCTGCCCAACAAGACCGCCACCTGCTG
GTCCCTGGACCCCGACCTGACCAACATCCTGGCCTCCTCCCGCTCCTACGCCATGCTGCTGTTC
GCCTGGGAGGGCTGGCACAACGCCGCCGGCATCCCCCTGAAGCCCCTGTACGAGGACTTCAC
CGCCCTGTCCAACGAGGCCTACAAGCAGGACGGCTTCACCGACACCGGCGCCTACTGGCGCT
CCTGGTACAACTCCCCCACCTTCGAGGACGACCTGGAGCACCTGTACCAGCAGCTGGAGCCC
CTGTACCTGAACCTGCACGCCTTCGTGCGCCGCGCCCTGCACCGCCGCTACGGCGACCGCTAC
ATCAACCTGCGCGGCCCCATCCCCGCCCACCTGCTGGGCGACATGTGGGCCCAGTCCTGGGA
GAACATCTACGACATGGTGGTGCCCTTCCCCGACAAGCCCAACCTGGACGTGACCTCCACCAT
GCTGCAGCAGGGCTGGAACGCCACCCACATGTTCCGCGTGGCCGAGGAGTTCTTCACCTCCCT
GGAGCTGTCCCCCATGCCCCCCGAGTTCTGGGAGGGCTCCATGCTGGAGAAGCCCGCCGACG
GCCGCGAGGTGGTGTGCCACGCCTCCGCCTGGGACTTCTACAACCGCAAGGACTTCCGCATCA
AGCAGTGCACCCGCGTGACCATGGACCAGCTGTCCACCGTGCACCACGAGATGGGCCACATC
CAGTACTACCTGCAGTACAAGGACCTGCCCGTGTCCCTGCGCCGCGGCGCCAACCCCGGCTTC
CACGAGGCCATCGGCGACGTGCTGGCCCTGTCCGTGTCCACCCCCGAGCACCTGCACAAGAT
CGGCCTGCTGGACCGCGTGACCAACGACACCGAGTCCGACATCAACTACCTGCTGAAGATGG
CCCTGGAGAAGATCGCCTTCCTGCCCTTCGGCTACCTGGTGGACCAGTGGCGCTGGGGCGTGT
TCTCCGGCCGCACCCCCCCCTCCCGCTACAACTTCGACTGGTGGTACCTGCGCACCAAGTACC
AGGGCATCTGCCCCCCCGTGACCCGCAACGAGACCCACTTCGACGCCGGCGCCAAGTTCCAC
GTGCCCAACGTGACCCCCTACATCCGCTACTTCGTGTCCTTCGTGCTGCAGTTCCAGTTCCACG
AGGCCCTGTGCAAGGAGGCCGGCTACGAGGGCCCCCTGCACCAGTGCGACATCTACCGCTCC
ACCAAGGCCGGCGCCAAGCTGCGCAAGGTGCTGCAGGCCGGCTCCTCCCGCCCCTGGCAGGA
GGTGCTGAAGGACATGGTGGGCCTGGACGCCCTGGACGCCCAGCCCCTGCTGAAGTACTTCC
AGCCCGTGACCCAGTGGCTGCAGGAGCAGAACCAGCAGAACGGCGAGGTGCTGGGCTGGCC
CGAGTACCAGTGGCACCCCCCCCTGCCCGACAACTACCCCGAGGGCATCGACCTGGTGACCG
ACGAGGCCGAGGCCTCCAAGTTCGTGGAGGAGTACGACCGCACCTCCCAGGTGGTGTGGAAC
GAGTACGCCGAGGCCAACTGGAACTACAACACCAACATCACCACCGAGACCTCCAAGATCCT
GCTGCAGAAGAACATGCAGATCGCCAACCACACCCTGAAGTACGGCACCCAGGCCCGCAAG
TTCGACGTGAACCAGCTGCAGAACACCACCATCAAGCGCATCATCAAGAAGGTGCAGGACCT
GGAGCGCGCCGCCCTGCCCGCCCAGGAGCTGGAGGAGTACAACAAGATCCTGCTGGACATG
GAGACCACCTACTCCGTGGCCACCGTGTGCCACCCCAACGGCTCCTGCCTGCAGCTGGAGCCC
GACCTGACCAACGTGATGGCCACCTCCCGCAAGTACGAGGACCTGCTGTGGGCCTGGGAGGG
CTGGCGCGACAAGGCCGGCCGCGCCATCCTGCAGTTCTACCCCAAGTACGTGGAGCTGATCA
ACCAGGCCGCCCGCCTGAACGGCTACGTGGACGCCGGCGACTCCTGGCGCTCCATGTACGAG
ACCCCCTCCCTGGAGCAGGACCTGGAGCGCCTGTTCCAGGAGCTGCAGCCCCTGTACCTGAA
CCTGCACGCCTACGTGCGCCGCGCCCTGCACCGCCACTACGGCGCCCAGCACATCAACCTGG
AGGGCCCCATCCCCGCCCACCTGCTGGGCAACATGTGGGCCCAGACCTGGTCCAACATCTAC
GACCTGGTGGTGCCCTTCCCCTCCGCCCCCTCCATGGACACCACCGAGGCCATGCTGAAGCAG
GGCTGGACCCCCCGCCGCATGTTCAAGGAGGCCGACGACTTCTTCACCTCCCTGGGCCTGCTG
CCCGTGCCCCCCGAGTTCTGGAACAAGTCCATGCTGGAGAAGCCCACCGACGGCCGCGAGGT
GGTGTGCCACGCCTCCGCCTGGGACTTCTACAACGGCAAGGACTTCCGCATCAAGCAGTGCA
CCACCGTGAACCTGGAGGACCTGGTGGTGGCCCACCACGAGATGGGCCACATCCAGTACTTC
ATGCAGTACAAGGACCTGCCCGTGGCCCTGCGCGAGGGCGCCAACCCCGGCTTCCACGAGGC
CATCGGCGACGTGCTGGCCCTGTCCGTGTCCACCCCCAAGCACCTGCACTCCCTGAACCTGCT
GTCCTCCGAGGGCGGCTCCGACGAGCACGACATCAACTTCCTGATGAAGATGGCCCTGGACA

AGATCGCCTTCATCCCCTTCTCCTACCTGGTGGACCAGTGGCGCTGGCGCGTGTTCGACGGCTC
CATCACCAAGGAGAACTACAACCAGGAGTGGTGGTCCCTGCGCCTGAAGTACCAGGGCCTGT
GCCCCCCCGTGCCCCGCACCCAGGGCGACTTCGACCCCGGCGCCAAGTTCCACATCCCCTCCT
CCGTGCCCTACATCCGCTACTTCGTGTCCTTCATCATCCAGTTCCAGTTCCACGAGGCCCTGTG
CCAGGCCGCCGGCCACACCGGCCCCCTGCACAAGTGCGACATCTACCAGTCCAAGGAGGCCG
GCCAGCGCCTGGCCACCGCCATGAAGCTGGGCTTCTCCCGCCCCTGGCCCGAGGCCATGCAG
CTGATCACCGGCCAGCCCAACATGTCCGCCTCCGCCATGCTGTCCTACTTCAAGCCCCTGCTG
GACTGGCTGCGCACCGAGAACGAGCTGCACGGCGAGAAGCTGGGCTGGCCCCAGTACAACT
GGACCCCCAACTCCGCCCGCTAACTCGAGTGCGGCCGC
