# Supplementary material for: Albumin-Bound Fatty Acids Modulate Endogenous Angiotensin-Converting Enzyme (ACE) Inhibition
Source: Biomedicines. 2026 Jan 4;14(1):103. doi: 10.3390/biomedicines14010103 (PMC12838124; doi:10.3390/biomedicines14010103)
Supplement: Supplementary file 1 [file biomedicines-14-00103-s001.zip › Supplementary Figure S2.pdf]

Supplementary Figure S2.

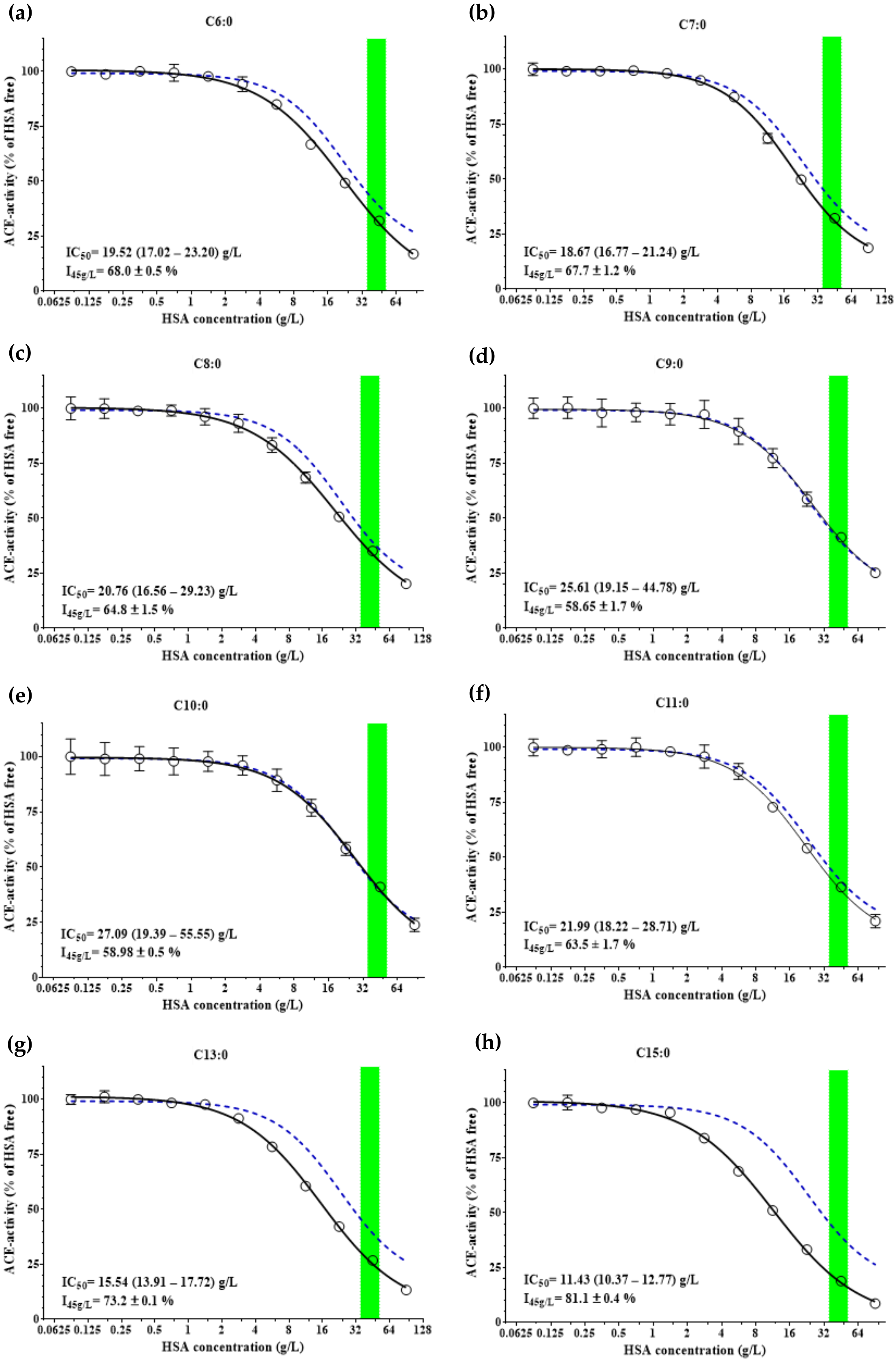

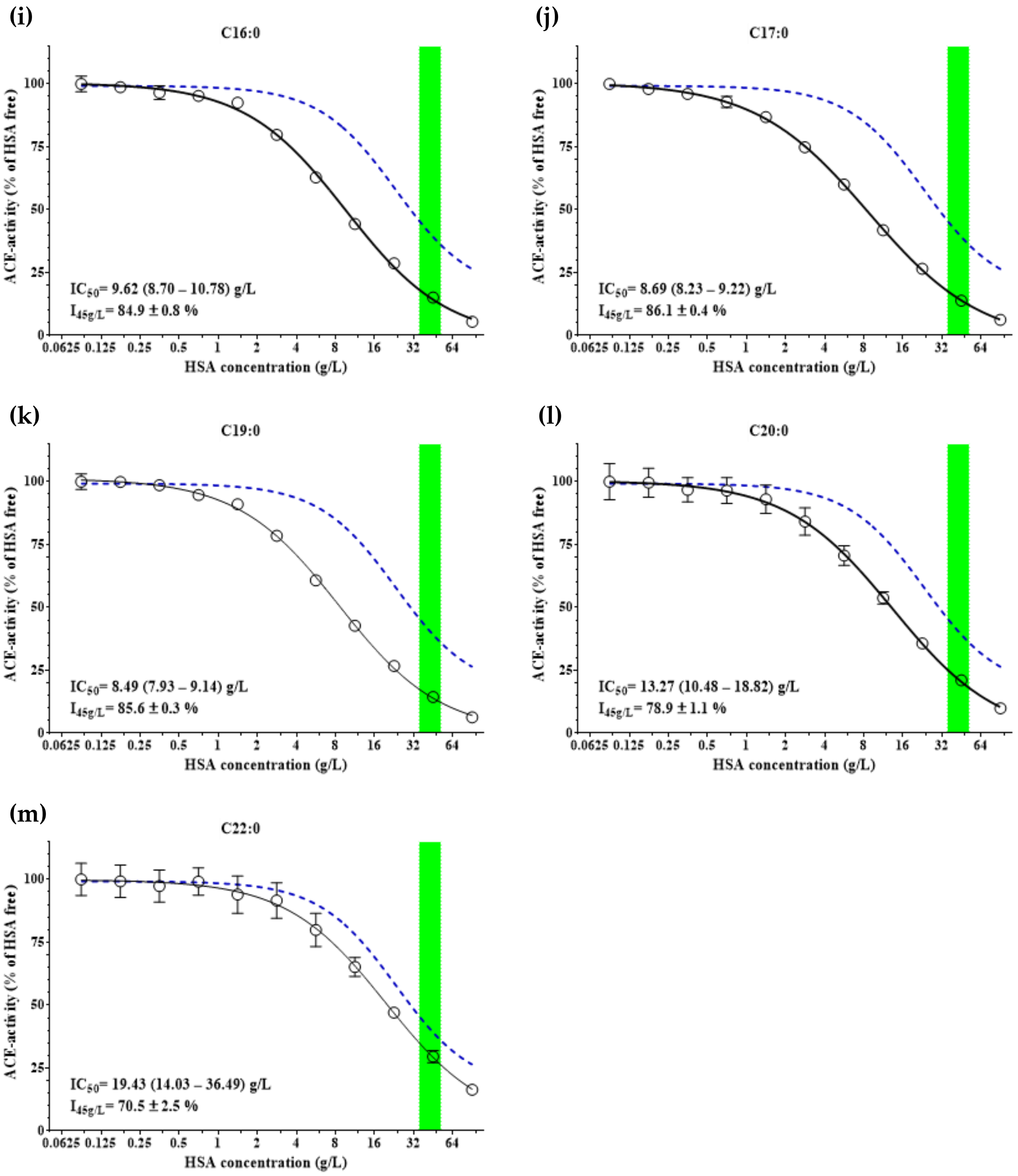

Supplementary Figure S2. Concentration–response curves illustrating the effect of saturated fatty acids of increasing carbon-chain length on the ACE-inhibitory capacity of HSA. Panels (a–m) show recombinant ACE activity measured in the presence of fatty-acid–free HSA (control, blue dashed line) or HSA pretreated with individual saturated FFAs ranging from C6:0 to C22:0 (black line). ACE activity is expressed as a percentage of HSA-free ACE activity. The corresponding four-parameter logistic inhibition curves are presented together with the calculated IC_{50} values (95% CI). Error bars represent the mean ± SD of three independent measurements. The green shaded band indicates the physiological serum HSA concentration range (35–52 g/L).
