# Supplementary material for: Albumin-Bound Fatty Acids Modulate Endogenous Angiotensin-Converting Enzyme (ACE) Inhibition
Source: Biomedicines. 2026 Jan 4;14(1):103. doi: 10.3390/biomedicines14010103 (PMC12838124; doi:10.3390/biomedicines14010103)
Supplement: Supplementary file 1 [file biomedicines-14-00103-s001.zip › Supplementary Table S1.pdf]

Supplementary Table S1.

Trivial name	*C:D (n–x)* nomenclature	Distributor	Catalogue number	Purity	LOT number	Manufacturing Country
**Caprylic acid**	C8:0	Merck	C2875	≥99%	MKBQ7766V	Malaysia
**Pelargonic acid**	C9:0	Merck	N5502	≥97%	055H0302V	USA
**Capric acid**	C10:0	Merck	C1875	≥98%	BCBJ3927V	Malaysia
**Undecylic acid**	C11:0	Merck	171476	98%	STBC5996V	India
**Lauric acid**	C12:0	Merck	L4250	≥99%	MKBQ9494V	Malaysia
**Tridecylic acid**	C13:0	Merck	T0502	≥98%	STBD5357V	Germany
**Myristic acid**	C14:0	Merck	70082	≥98%	BCBM4636V	Germany
**Myristoleic acid**	C14:1 (n-5)	Merck	M3525	≥99%	SLBR0892V	USA
**Pentadecylic acid**	C15:0	Merck	P6125	~99%	SHBC9557V	USA
**Palmitic acid**	C16:0	Merck	P0500	≥99%	SLBJ3296V	Malaysia
**Palmitoleic acid**	C16:1 (n-7)	Merck	P9417	≥98%	SLBQ4106V	USA
**Margaric acid**	C17:0	Merck	H3500	≥98%	MKBR1534V	Japan
**Stearic acid**	C18:0	Merck	S4751	≥98,5%	BCBM1870V	Switzerland
**Oleic acid**	C18:1 (n-9)	Merck	O1008	≥99%	SLBQ7857V	India
**cis-Vaccenic acid**	C18:1 (n-7)	Merck	V0384	≥97%	SLBP9321V	USA
**Vaccenic acid**	C18:1 (n-7)	Merck	V1131	≥99%	MKBT3619V	USA
**Linoleic acid**	C18:2 (n-6)	Merck	L1376	≥99%	SLBF3501V	USA
**α-Linolenic acid**	C18:3 (n-3)	Merck	L2376	≥99%	SLBQ1668V	USA
**γ-Linolenic acid**	C18:3 (n-6)	Merck	L2378	100%	MKBW3207V	USA
**Nonadecylic acid**	C19:0	Merck	72332	100%	BCBM8951V	Switzerland
**Arachidic acid**	C20:0	Merck	10930	≥99%	1373317V	Switzerland
**Gondoic acid**	C20:1 (n-9)	Merck	E3635	≥99%	MKBW8741V	USA
**Dihomolinoleic acid**	C20:2 (n-6)	Merck	E3127	≥98%	SLBC1978V	USA
**Arachidonic acid**	C20:4 (n-6)	Merck	A3611	≥98,5%	SLBP7568V	USA
**Eicosapentaenoic acid (Timnodonic acid)**	C20:5 (n-3)	Merck	E7006	≥85%	SLBL9041V	USA
**Behenic acid**	C22:0	Merck	216941	99%	MKBP9612V	USA
**Erucic acid**	C22:1 (n-9)	Merck	E3385	≥99%	107K5206V	USA
**Docosahexaenoic acid (Cervonic acid)**	C22:6 (n-3)	Merck	D2534	≥98%	SLBQ4029V	USA
**Nervonic acid**	C24:1 (n-9)	Merck	N1514	≥99%	MKBZ1162V	USA

Supplementary Table S1. Characteristics of the fatty acids examined in this study. The table lists each compound's trivial name, carbon-chain nomenclature (C:D, n-x), distributor, catalogue number, reported purity, LOT number, and country of manufacture. All reagents were obtained from Merck and used without further purification.
